# Titration of Basal and Prandial Insulin Doses With the Initiation of Glucagon-Like Peptide-1 Receptor Agonist Therapy

**DOI:** 10.7759/cureus.59899

**Published:** 2024-05-08

**Authors:** Alexander C Hill, Prasanna Santhanam, Caroline B Samples, Roberto Mitsui Akagi, Tariq Latif, Rodhan Khthir

**Affiliations:** 1 Department of Clinical Pharmacy, Marshall Health, Huntington, USA; 2 Division of Endocrinology Diabetes and Metabolism, Johns Hopkins University School of Medicine, Baltimore, USA; 3 Department of Internal Medicine, Marshall Health, Huntington, USA; 4 Department of Endocrinology, Diabetes, and Metabolism, Sanford Health, Bismarck, USA

**Keywords:** diabetes type 2, insulin-dependent diabetes mellitus, insulin regimen, insulin requirement, glucagon-like peptide-1 receptor agonists

## Abstract

Objective

Glucagon-like peptide-1 receptor agonists (GLP-1 RAs) have demonstrated significant efficacy in improving glycemic control in type 2 diabetes mellitus, which often results in decreased insulin dose requirements. The purpose of this study was to examine the changes in basal and prandial insulin dose requirements from baseline to three months following initiation of a GLP-1 RA.

Methodology

A retrospective chart review was conducted of adult insulin-treated patients at the Chertow Diabetes Center, Huntington, WV, who were started on GLP-1 RAs for 24 months.

Results

Mean daily basal insulin doses decreased by 8.7 units (*P* = 0.29; mean 8.3% change) and mean daily prandial insulin doses decreased by 9.4 units (*P* = 0.10; mean 18.4% change) from baseline to three months after starting a GLP-1 RA. Average hemoglobin A1c significantly decreased from 8.8% (73 mmol/mol) at baseline to 8.0% (64 mmol/mol) at three months (*P* < 0.001). Significant decreases from baseline to three months were also observed in mean body weight, mean low-density lipoprotein (LDL) cholesterol, and mean total cholesterol.

Conclusions

GLP-1 RA therapy was associated with a significant decrease in hemoglobin A1c, body weight, and LDL-cholesterol from baseline to three months after initiation. Therapy with GLP-1 RAs was also associated with an overall decrease in daily basal and prandial insulin dose requirements, although this finding did not reach statistical significance.

## Introduction

Glucagon-like peptide-1 receptor agonists (GLP-1 RAs) considerably improve glycemic control by improving insulin secretion in a glucose-dependent way and decreasing glucagon secretion, causing an average hemoglobin A1c reduction of approximately 1.2% [1]. These agents have also demonstrated several other benefits in addition to decreasing blood glucose levels. Through delaying gastric emptying and consequently decreasing appetite, GLP-1 RAs can promote weight loss [1-2]. Studies have also demonstrated positive effects on blood pressure and cholesterol levels. A meta-analysis of 60 trials showed that, on average, GLP-1 RAs were associated with a positive effect on blood pressure [3]. Another meta-analysis of 35 trials showed that GLP-1 RAs were associated with significant reductions in low-density lipoprotein (LDL, about 3.1 mg/dL) and total cholesterol (about 5 mg/dL) [4]. Another benefit of GLP-1 RAs is a lower risk of hypoglycemia when used as monotherapy compared with many other antidiabetic agents, such as sulfonylureas [5].

Combining a GLP-1 RA and insulin is an appealing treatment strategy for type 2 diabetes because of its potential for achieving substantial improvements in glycemic control with a relatively low risk of hypoglycemia or weight gain. A meta-analysis that examined randomized controlled trials found that combination treatment with GLP-1 RAs and basal insulin led to a mean greater reduction in A1c compared with any other anti-diabetic treatment strategy (*P* = 0.0047) [3]. Additionally, there was no difference found in the relative risk of hypoglycemia with GLP-1 RA and basal insulin combination treatment compared with other treatments.

Because of their potent blood glucose-lowering efficacy, the effect of GLP-1 RAs on insulin requirements in insulin-treated patients can be extremely variable and represent a challenge, especially in patients who are receiving high daily doses of insulin. A randomized controlled trial compared exenatide 10 mcg twice daily to placebo in type 2 diabetic patients for 30 weeks, with both interventions given concomitantly with basal insulin [6]. At randomization, patients with a hemoglobin A1c greater than 8% (64 mmol/mol) continued to receive their current insulin doses, while patients with an A1c less than 8% (64 mmol/mol) decreased their insulin doses by 20%. At 30 weeks, the reduction of A1c was greater in the exenatide group than placebo (*P* < 0.001).

The prescribing information for all currently commercially available GLP-1 RAs states that patients may need a decrease in insulin doses upon initiation of a GLP-1 RA to avoid hypoglycemia [7-13]. However, there are insufficient data on appropriate methods for titrating and decreasing insulin doses upon GLP-1 RA initiation [1]. The purpose of this study was to examine the changes in basal and prandial insulin requirements within the first three months following initiation of GLP-1 RAs.

## Materials and methods

Study design

A retrospective chart review using patients’ electronic medical records was conducted on adult insulin-treated patients who were started on GLP-1 RAs over 24 months at the Chertow Diabetes Center, Huntington, WV. Patient weight, hemoglobin A1c, blood pressure, serum lipids, and basal and prandial insulin daily doses were all collected at baseline before initiation of GLP-1 RA and after three months of therapy. Other baseline characteristics collected before initiation of GLP-1 RA included glomerular filtration rate (GFR) and C-peptide level. Changes in basal and prandial insulin dose requirements, hemoglobin A1c, weight, blood pressure, and serum lipids were calculated by comparing the data from the visit before starting the GLP-1 RA to the following visit (3-12 months from baseline data).

Patients were included if they had a diagnosis of type 1 or type 2 diabetes and were over the age of 18 years. Patients were excluded if they stopped the GLP-1 RA after less than four weeks from initiation. This study was approved by the institutional review board of Marshall University.

When patients were started on GLP-1 RAs, the clinical pharmacist was available for consultation, which could include counseling on the new medication and ensuring insurance coverage and affordability of the GLP-1 RA. Consultation services could also include diabetes therapy management following GLP-1 RA initiation, which involved the clinical pharmacist following up with patients regularly to monitor their blood glucose readings and then consult with the patient's provider to make any appropriate therapy changes. Referral to clinical pharmacist consultation was based on provider discretion; for example, providers would often refer patients if they felt they needed closer monitoring of response and need for insulin dose adjustments after starting GLP-1 RA therapy.

Outcome measures

The primary outcome of this study was the change in insulin dose requirements from baseline to three months after GLP-1 RA initiation. Secondary outcomes were the changes in hemoglobin A1c, weight, blood pressure, and serum lipids from baseline to three months after GLP-1 RA initiation.

Statistical analyses

Statistical analysis was performed using SPSS version 18 (SPSS Inc., Chicago), a statistical software, frequently employed in biomedical sciences [14]. We also used the R base package for data cleaning, cachenation, and tabulation [15]. Mediation analysis is a tool for statistical inferences, widely applied recently in psychological, social sciences [16]. SPSS has a macro named PROCESS that we employed for the mediation analysis [17]. It is used for estimation of indirect, direct, and complete effects with categorical independent variables [18]. We used mediation analysis in this study to examine these effects on short-acting insulin doses following initiation of GLP-1 RA. A paired t-test was used to compare daily basal and prandial insulin doses, hemoglobin A1c, weight, blood pressure, and serum lipids before and three months following GLP-1 RA initiation. Statistical significance was set at a *P*-value less than or equal to 0.05.

## Results

Study population

Initially, 135 patients were screened for eligibility, with 83 included in the final analysis. A total of 52 patients were not included, with most not included because they were not receiving insulin therapy. Population baseline characteristics are listed in Table 1.

**Table 1:** Population baseline characteristics. HDL-C, high-density lipoprotein cholesterol; LDL-C, low-density lipoprotein cholesterol

Characteristics		Value
Age (years), mean (SD)		57 (11.4)
Gender, number (%)	Female	42 (50.6)
	Male	41 (49.4)
Duration of diabetes (years), mean (SD)		12.8 (9.3)
Managed by a pharmacist, number (%)		26 (31)
Hemoglobin A1c, mean [SD], % (mmol/mol)		8.8 (73) [1.6]
Body weight (kg), mean (SD)		119.9 (32.6)
C-peptide (ng/mL), mean (SD)		4.6 (2.8)
Blood pressure (mmHg), mean (SD)	Systolic	128 (13.3)
	Diastolic	75 (8.6)
Serum lipids (mg/dL), mean (SD)	HDL-C	41 (11.5)
	LDL-C	90 (34.9)
	Total cholesterol	166 (40.6)
	Triglycerides	197 (105.7)
Glomerular filtration rate, number (%)	>60 mL/min	47 (56.5)
	30-59 mL/min	29 (34.9)
	<30 mL/min	5 (6.0)

Insulins were categorized as basal or prandial. Insulin lispro and aspart were categorized as prandial insulins, while insulin regular U-500, glargine, Neutral Protamine Hagedorn (NPH), detemir, and degludec were classified as basal insulins. The distribution of GLP-1 RAs received by patients is detailed in Table 2. Most patients received semaglutide and dulaglutide.

**Table 2:** GLP-1 RAs received. GLP-1 RA, glucagon-like peptide-1 receptor agonist

Agent	Number of patients (%)
Dulaglutide	18 (21.7)
Exenatide	1 (1.2)
Exenatide, extended release	1 (1.2)
Liraglutide	3 (3.6)
Semaglutide	60 (72.3)

Outcome measures

Insulin doses before and three months after starting GLP-1 RA therapy are detailed in Table 3. Mean daily basal and prandial insulin doses both decreased from baseline to three months following GLP-1 RA initiation, by 8.7 units (*P* = 0.29; mean 8.3% change) and by 9.4 units (*P* = 0.10; mean 18.4% change), respectively.

**Table 3:** Primary outcome results.

Type of insulin	Initial daily dose, mean, units	Daily dose at three months, mean, units	Dose change from baseline, mean (SD), units	Percentage change from baseline, mean	*P*-value
Basal	88.2	79.5	–8.7 (71.7)	8.3	0.29
Prandial	57.2	49.6	–9.4 (39.0)	18.4	0.10

Results of the secondary outcomes are detailed in Table 4. Average hemoglobin A1c, body weight, LDL cholesterol, and total cholesterol all significantly decreased from baseline to three months following GLP-1 RA initiation. Systolic and diastolic blood pressure, HDL cholesterol, and triglyceride levels all decreased as well, but these results did not meet statistical significance.

**Table 4:** Secondary outcomes results. HDL, high-density lipoprotein; LDL, low-density lipoprotein

Variable	Baseline	Three months	*P*-value
Hemoglobin A1c (mmol/mol), mean [SD], %	8.8 [1.6] (73)	8.0 [1.2] (64)	<0.001
Body weight (kg), mean (SD)	119.9 (32.6)	116.8 (29.8)	<0.001
Systolic blood pressure (mmHg), mean (SD)	127.5 (13.3)	124.8 (14.4)	0.21
Diastolic blood pressure (mmHg), mean (SD)	75.2 (8.6)	73.5 (8.5)	0.19
HDL cholesterol (mg/dL), mean (SD)	41.5 (11.5)	40.7 (10.1)	0.96
LDL cholesterol (mg/dL), mean (SD)	89.9 (34.9)	75.5 (40.0)	0.04
Total cholesterol (mg/dL), mean (SD)	165.9 (40.6)	150.5 (37.5)	0.03
Triglycerides (mg/dL), mean (SD)	200.4 (105.7)	197.3 (100.1)	0.57

Results of the mediation analysis to examine the total, direct, and indirect effects on short-acting insulin doses following initiation of GLP-1 RA are detailed in Table 5.

**Table 5:** Results of mediation analysis: total, direct, and indirect effects on short-acting insulin doses.

Outcome variable		Effect
Body weight	Total effect	0.6835
	Direct effect	0.6275
	Indirect effect	0.0560
Hemoglobin A1c, Initial	Total effect	0.6608
	Direct effect	0.6806
	Indirect effect	-0.0198
Hemoglobin A1c, Final	Total effect	0.6385
	Direct effect	0.6360
	Indirect effect	0.0025

## Discussion

In this study, average daily doses of both basal and prandial insulin were found to decrease in the three months following GLP-1 RA initiation. Although this finding was not statistically significant, mean hemoglobin A1c levels were found to decrease significantly over this period as well, despite the overall decreases in insulin doses. Additionally, average body weight, LDL cholesterol, and total cholesterol levels all decreased significantly after starting GLP-1 RAs. Decreases in blood pressure and serum triglyceride levels were also observed, but these changes were not statistically significant.

The baseline demographics assessed for the study population notably included C-peptide values. These were collected to assess and report the overall incidence of type 1 versus type 2 diabetes diagnosis in our study population so that the results could be interpreted with this taken into account. Additionally, a preliminary goal of this study was to assess if patients with higher C-peptide values would have more reduction in insulin requirements; however, the sample size was too small to draw any conclusion about this. 

One strength of this study was a similar number of male and female patients, making the results applicable to both genders. Additionally, the study examined a novel primary outcome that has not sufficiently been studied previously. Another strength of this study is the use of real-world data. While this may sacrifice the ability to definitively demonstrate causality due to the number of possible confounders, the findings can be considered clinically applicable with external validity.

A limitation of this study was its retrospective design - investigators could not control all of the variables that could affect study outcomes. Additionally, the study had a small sample size, which may have played a role in some of the results being found non-statistically significant. Another limitation of this study was missing data points for some variables. As some patients were seen by a provider less frequently than every three months, some variables, such as serum lipid levels, were not available or may have been collected further than three months following GLP-1 RA initiation. Regarding serum lipid levels, another limitation of this study was that documentation of statin use was not consistently available for study subjects. Therefore, this data was not collected, so any influence this may have had on serum lipid levels in addition to GLP-1 RA therapy is unknown. Finally, this study had a relatively short duration. Since many GLP-1 RAs need dose titrations for several weeks, these agents may not have been at a steady state for the full three-month period, so the full therapeutic effects of the medications may not have been realized at the scheduled follow-up.

A majority of the patients in this study were started on semaglutide as their GLP-1 RA, likely because it is a preferred agent on most insurance plans in West Virginia. Additionally, with its once-weekly dosing, the risk for noncompliance is relatively low. Renal function measured as GFR was reported for study subjects to provide a rationale behind why some GLP-1 RA agents (e.g., exenatide), which have recommended dose adjustments for patients with renal impairment, were used less frequently than others (e.g., semaglutide and dulaglutide), which do not have any dose adjustments recommended for patients with renal impairment. However, the high percentage of semaglutide use could potentially limit the applicability of the study results to other GLP-1 RAs.

## Conclusions

While the finding did not meet statistical significance, total daily basal and prandial insulin doses decreased after initiation of a GLP-1 RA. Hemoglobin A1c decreased significantly following GLP-1 RA initiation despite the decreases in insulin dose requirements. Based on these promising results, more studies with longer durations and larger sample sizes are indicated.

## References

[1] Artigas CF, Stokes V, Tan GD, Theodorakis MJ (2015). Insulin dose adjustments with add-on glucagon-like peptide-1 receptor (GLP-1R) agonists in clinical practice. Expert Opin Pharmacother.

[2] Anderson SL, Trujillo JM (2016). Basal insulin use with GLP-1 receptor agonists. Diabetes Spectr.

[3] Eng C, Kramer C, Zinman B (2014). Glucagon-like peptide-1 receptor agonist and basal insulin combination treatment for the management of type 2 diabetes: a systematic review and meta-analysis. Lancet.

[4] Sun F, Wu S, Wang J (2015). Effect of glucagon-like peptide-1 receptor agonists on lipid profiles among type 2 diabetes: a systematic review and network meta-analysis. Clin Ther.

[5] Pinkney J, Fox T, Ranganath L (2010). Selecting GLP-1 agonists in the management of type 2 diabetes: differential pharmacology and therapeutic benefits of liraglutide and exenatide. Ther Clin Risk Manag.

[6] Buse JB, Bergenstal RM, Glass LC (2011). Use of twice-daily exenatide in basal insulin-treated patients with type 2 diabetes: a randomized, controlled trial. Ann Intern Med.

[7] DailyMed DailyMed (2022). Ozempic® (Semaglutide). https://dailymed.nlm.nih.gov/dailymed/drugInfo.cfm?setid=adec4fd2-6858-4c99-91d4-531f5f2a2d79.

[8] DailyMed DailyMed (2022). Victoza® (Liraglutide). https://dailymed.nlm.nih.gov/dailymed/drugInfo.cfm?setid=5a9ef4ea-c76a-4d34-a604-27c5b505f5a4.

[9] DailyMed DailyMed (2022). Byetta® (Exenatide).

[10] DailyMed DailyMed (2022). Bydureon® (Exenatide). https://dailymed.nlm.nih.gov/dailymed/drugInfo.cfm?setid=71fe88be-b4e6-4c2d-9cc3-8b1864467776.

[11] DailyMed DailyMed (2022). Trulicity® (Dulaglutide). https://dailymed.nlm.nih.gov/dailymed/drugInfo.cfm?setid=463050bd-2b1c-40f5-b3c3-0a04bb433309.

[12] DailyMed DailyMed (2022). Adlyxin® (Lixisenatide). LLC.

[13] DailyMed DailyMed (2022). Rybelsus® (Semaglutide). https://dailymed.nlm.nih.gov/dailymed/drugInfo.cfm?setid=27f15fac-7d98-4114-a2ec-92494a91da98.

[14] Kremelberg D (2010). Practical Statistics: A Quick and Easy Guide to IBM® SPSS® Statistics, STATA, and Other Statistical Software.

[15] Culpepper SA, Aguinis H (2011). R is for revolution: a cutting-edge, free, open source statistical package. Organ Res Methods.

[16] Valeri L, Vanderweele TJ (2013). Mediation analysis allowing for exposure-mediator interactions and causal interpretation: theoretical assumptions and implementation with SAS and SPSS macros. Psychol Methods.

[17] Hayes AF: PROCESS (2022). PROCESS: A versatile computational tool for observed variable mediation, moderation, and conditional process modeling [White paper]. http://www.afhayes.com/public/process2012.pdf.

[18] Hayes AF, Preacher KJ (2014). Statistical mediation analysis with a multicategorical independent variable. Br J Math Stat Psychol.

